# Near Infrared Imaging of EGFR of Oral Squamous Cell Carcinoma in Mice Administered Arsenic Trioxide

**DOI:** 10.1371/journal.pone.0046255

**Published:** 2012-09-28

**Authors:** Lingbo Zhang, Kezheng Wang, Falin Zhao, Weiping Hu, Junjie Chen, Gregory M. Lanza, Baozhong Shen, Bin Zhang

**Affiliations:** 1 Stomatology Department, Institute of Hard Tissue Development and Regeneration, 2nd Affiliated Hospital, Harbin Medical University, Harbin, Heilongjiang, China; 2 Radiology Department and Molecular Imaging Center, 4th Affiliated Hospital, Harbin Medical University, Harbin, Heilongjiang, China; 3 School of Health Management, Hangzhou Normal University, Hangzhou, Zhejiang, China; 4 Division of Cardiology and C-TRAIN, Washington University School of Medicine, St. Louis, Missouri, United States of America; National Institute of Health, United States of America

## Abstract

**Background:**

The effectiveness of near-infrared imaging (NIR) interrogation of epidermal growth factor receptor (EGFR) expression as a sensitive biomarker of oral squamous cell carcinoma (OSCC) response to arsenic trioxide therapy was studied in mice.

**Material and Methods:**

A431 OSCC *in vitro* were exposed to 0 µM, 0.5 µM, 2.5 µM, or 5 µM of As_2_O_3_ for 0 h, 24 h, 48 h and 72 h. Confocal microscopy and flow cytometry confirmed EGFR expression and demonstrated a sensitivity dose-related signal decline with As_2_O_3_ treatment. Next, mice with pharynx-implanted A431 cells received As_2_O_3_ i.p. every 48 h at 0.0, 0.5, 2.5, or 5 mg/kg/day (n = 6/group) from day 0 to 10. An intravenous NIR probe, EGF-Cy5.5, was injected at baseline and on days 4, 8, and 12 for dynamic NIR imaging. Tumor volume and body weights were measured three times weekly.

**Results:**

*In vitro*, A431 EGFR expression was well appreciated in the controls and decreased (*p*<0.05) with increasing As_2_O_3_ dose and treatment duration. *In vivo* EGFR NIR tumor signal intensity decreased (*p*<0.05) in As_2_O_3_ treated groups versus controls from days 4 to 12, consistent with increasing dosage. Tumor volume diminished in a dose-related manner while body weight was unaffected. Immunohistochemical staining of excised tumors confirmed that EGFR expression was reduced by As_2_O_3_ treatment in a dose responsive pattern.

**Conclusion:**

This study demonstrates for the first time that OSCC can be interrogated *in vivo* by NIR molecular imaging of the EGFR and that this biomarker is effective for the longitudinal assessment of OSCC response to As_2_O_3_ treatment.

## Introduction

Oral squamous cell carcinoma (OSCC) is one of the ten most common cancers [1], and is by far the most common malignant neoplasm in the oral cavity [2]. Despite advances in diagnosis and therapy over the last three decades, the prognosis of OSCC remains unsatisfying, with increasing high rates of relapse and lymph node metastases. The overall five-year relative survival rate remains less than 60% [3]. Surgical therapy is the primary treatment for OSCC. However, only a minority of patients benefit from curative surgery, since most post-surgical patients succumb to locally advanced or metastatic disease and many suffer from marked facial disfigurement. Effective medical therapy to better debulk or cure OSCC would be preferred, particularly for patients not suitable for surgical resection. Unfortunately, OSCCs are resistant to most conventional chemotherapeutic drugs.

Arsenic trioxide (As_2_O_3_, TRISENOX) is the most widely used and studied arsenic-based anticancer drug [4]. It is an effective chemotherapeutic agent for treating relapsed or refractory acute promyelocytic leukemia (APL) [5]–[7]. Abundant preclinical evidence has shown that As_2_O_3_ is also effective on solid tumors in liver [8], lung [9], ovary [10], gastric system [11], prostate [12], nasopharynx [13], as well as the oral cavity [14]. However, the anticancer mechanisms of As_2_O_3_ for inhibiting growth and triggering apoptosis of cancer cells are not fully understood [15]. Recently, As_2_O_3_ was reported to inhibit epidermal growth factor receptor (EGFR) expression on the surface of OSCC cells in culture [16].

EGFR is an important biomarker and useful prognostic indicator in oral cancer [17], being widely overexpressed in dysplasia and OSCC [18], [19]. In OSCC patients, high EGFR expression is usually associated with poor prognosis [1], [18]. However, the *in vivo* therapeutic effect of As_2_O_3_ on EGFR expression of OSCC tumor xenografts has not been reported.

Rapid and direct interrogation of biomarkers to risk stratify and guide OSCC in patients would have significant clinical advantage [20]. Currently, immunohistochemistry (IHC) [2] and mRNA expression [21] are commonly used to assess EGFR expression at protein and gene levels in biopsy samples. Unfortunately, these *ex vivo* methods cannot provide quantitative and spatial information about OSCC EGFR expression *in situ* [22]. Noninvasive molecular imaging techniques for quantitatively assessing tumor biochemical status [23] based on quantitative EGFR imaging would facilitate initial *in vivo* selection of therapeutic carepaths and provide a tool for longitudinal monitoring of early recurrence [24]. Near infrared (NIR) fluorescence imaging of EGFR is well suited to evaluation of oral cavity lesions [25], [26], given the superficial nature of oral epithelial and submusosal cancers.

The goal of this study was to explore the feasibility of noninvasively monitoring the therapeutic effect of arsenic trioxide on EGFR expression of OSCC with NIR optical imaging *in vivo*.

## Results

### *In vitro* results with confocal microscopy, immunohistochemistry and flow cytometry

**Figure 1:**
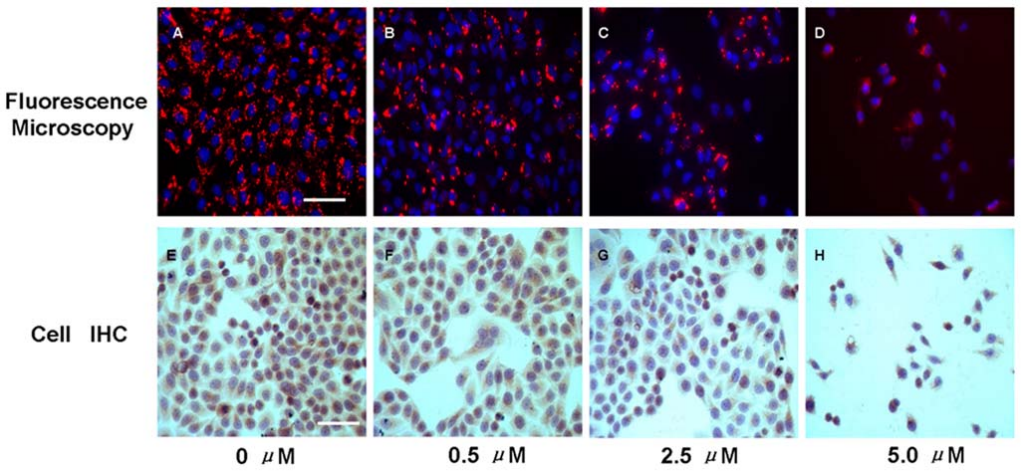
Effects of As_2_O_3_ on A431 cell EGFR expression. Cells were exposed to different concentrations of As_2_O_3_. At 48 h post-treatment, cells were assessed by fluorescence microscopy for visualization of the intake of EGF-Cy5.5 and cell immunohistochemistry for assay of EGFR expression. A–D, representative fluorescence images of different groups (Scale bar = 100 µm), Cy5.5 was pseudo-colored red, DAPI was pseudo-colored blue; E-H, representative images of cellular EGFR Immunohistochemistry assay (Scale bar = 100 µm), diaminobenzidine (DAB) showed as brown color represented EGFR expression and hematoxylin showed as blue color indicated the cellular nuclear. Interestingly, cell numbers decreased as the arsenic trioxide concentration increased from 0 µM to 5 µM in both fluorescent and immunostained images, indicating that As_2_O_3_ induced a dose-dependent inhibition on tumor cell proliferation as previously reported [31].

Fluorescence microscopy of targeted A-431 tumor cells (Figure 1, A–D) confirmed probe uptake into the cell membrane and cytoplasm. Less intense cellular fluorescence signal was observed in all As_2_O_3_ treated cells when compared with control cells. Additionally, fluorescent signal intensity decreased in the cells receiving higher As_2_O_3_ concentrations (2.5 µM or 5.0 µM) compared with those treated with 0.5 µM. The change in optical contrast was corroborated with the levels of EGFR expression (Figure 1, E–H) appreciated with immunohistochemistry microscopy.

**Figure 2:**
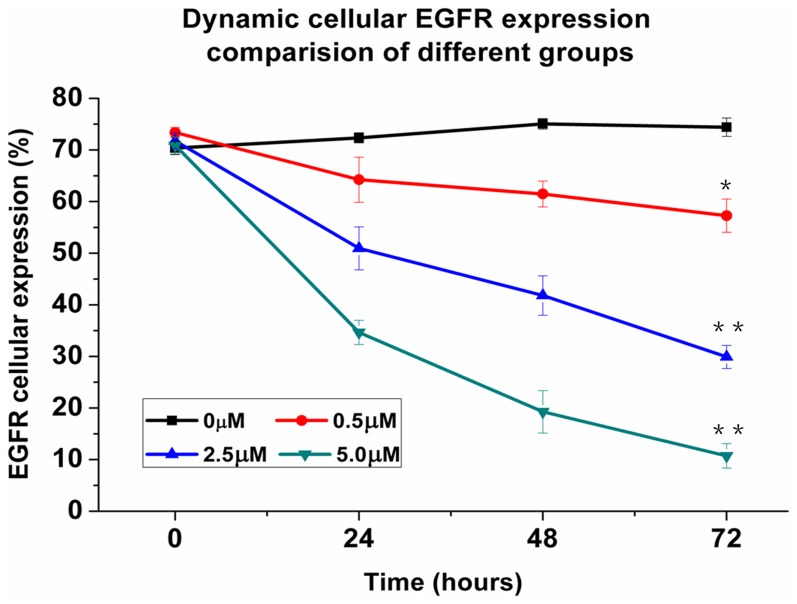
The cellular EGFR expression percentage of different treatment groups *in vitro* assessed by flow cytometry. The dynamic EGFR cellular expression percentage after treatment of different concentrations of As_2_O_3_ (0 µM, 0.5 µM, 2.5 µM, 5.0 µM) varied with time (*p<0.05, **p<0.01). All experiments were carried out in triplicate; each point represents the mean ± standard error values.

Using flow cytometry, the EGFR expression in treatment group decreased from 0 h, 24 h, 48 h, to 72 h while in the control group, EGFR expression increased over the same time course (Figure 2). Before treatment, cellular EGFR expression of 0.0 µM 0.5 µM, 2.5 µM and 5.0 µM groups were 70.4±1.3%, 73.4±1.0%, 71.8±1.5%, and 70.9±1.7% (n = 3, *p*>0.05 for all comparisons). At 72 h post treatment, cellular EGFR expression was 57.3±3.2% (*p*<0.05), 29.9±2.2% (*p*<0.01), and 10.7±2.4% (*p*<0.01) in cells treated with 0.5 µM, 2.5 µM, and 5.0 µM As_2_O_3_, respectively, which were significant lower than the control group (74.4±1.8%, * *p*<0.05, ** *p*<0.01).

### *In vivo* NIR imaging of tumor response to As_2_O*_3_*

**Figure 3:**
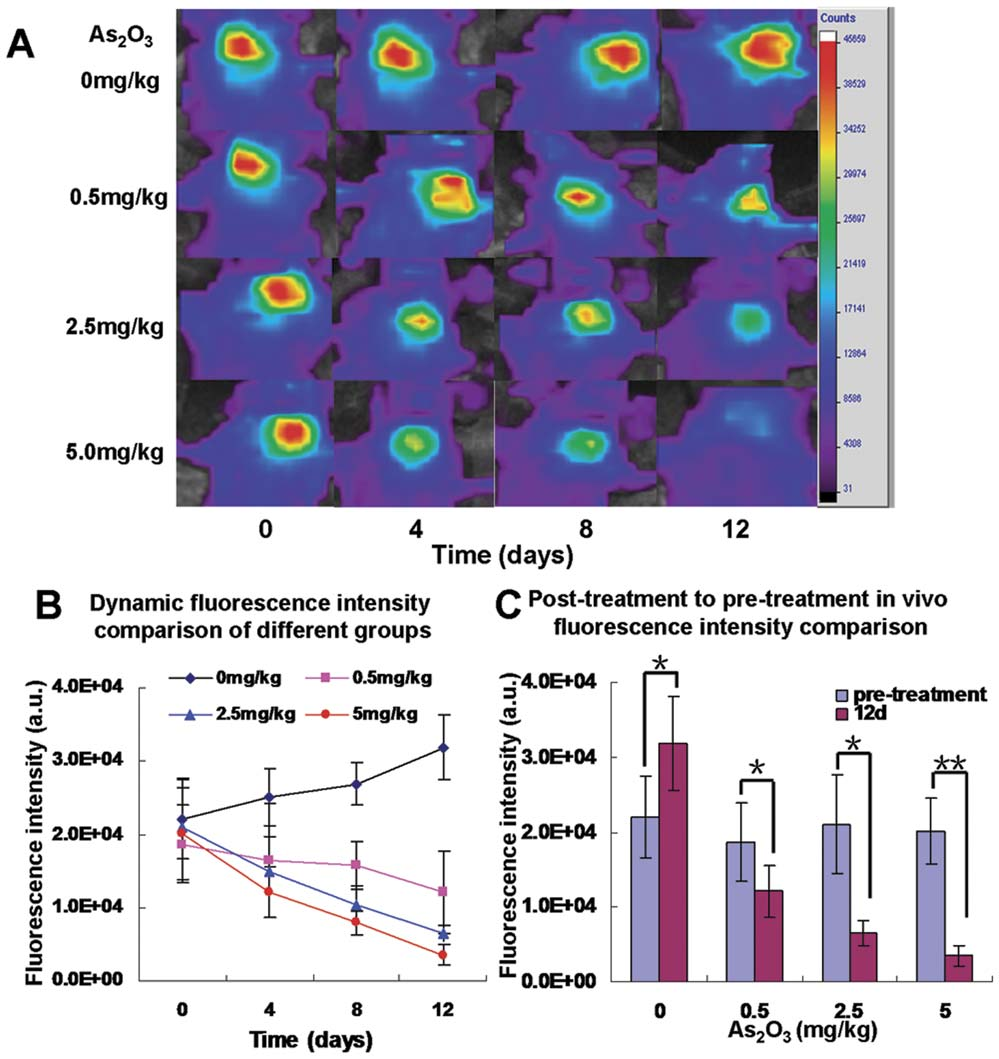
*In vivo* dynamic near-infrared fluorescent imaging of A-431 tumor models. A: The representative fluorescence images of the tumor regions in mice were acquired at 4 h post injection of EGF-Cy5.5. Fluorescence signal from Cy5.5 was pseudo-colored red. B: The dynamic measurement comparison of fluorescence intensity of tumor in different groups. It was demonstrated that the fluorescence intensity in the tumor regions were changed with time (*p*<0.05). On day 0 (before As_2_O_3_ treatment), there was no significant difference of signal intensity of tumors between treatment and control groups (*p*>0.05). On day 4, 8, 12 (after As_2_O_3_ treatment), the signal intensity of EGF-Cy5.5 uptake by control group (0 mg/kg/day As_2_O_3_) gradually increased, while the intensities in other three groups with different concentrations (0.5 mg/kg/day, 2.5 mg/kg/day, 5.0 mg/kg/day) of As_2_O_3_ treatment gradually decreased (*p*<0.05). C: The *in vivo* fluorescence intensity was compared between post-treatment (on day 12) in four different groups compared with respective pre-treatment (on day 0). All plots are representative of results from groups of mice treated under the same experimental conditions. Each point represents the mean values (n = 6/group, *p<0.05, **p<0.01).

*In vivo* NIR fluorescent imaging of the tumor region of interest (ROI) was performed dynamically and fluorescence luminosity (signal intensity, SI) was measured before treatment (day 0) and 4, 8, 12 days after titrated As_2_O_3_ treatment (Figure 3A). On day 0 (before As_2_O_3_ treatment), the SI of the tumor ROIs among four groups (0.0 mg/kg/day, 0.5 mg/kg/day, 2.5 mg/kg/day, 5.0 mg/kg/day) were not different: (2.20±0.54)×10^4^ a.u., (1.87±0.53)×10^4^ a.u., (2.10±0.66)×10^4^ a.u. and (2.01±0.44)×10^4^ a.u., respectively (Figure 3B, *p*>0.05). From days 4 to 12, tumor SI increased progressively in the control group but gradually decreased in As_2_O_3_ groups. On day 12, the tumor SI was decreased inversely with each As_2_O_3_ dosage (1.21±0.35)×10^4^ a.u., (0.65±0.16)×10^4^ a.u., and (0.35±0.14)×10^4^ a.u., respectively, versus the control group (3.18±0.63)×10^4^ a.u. (*p*<0.05, Figure 3C).

### Inhibition of tumor growth by arsenic trioxide

**Figure 4:**
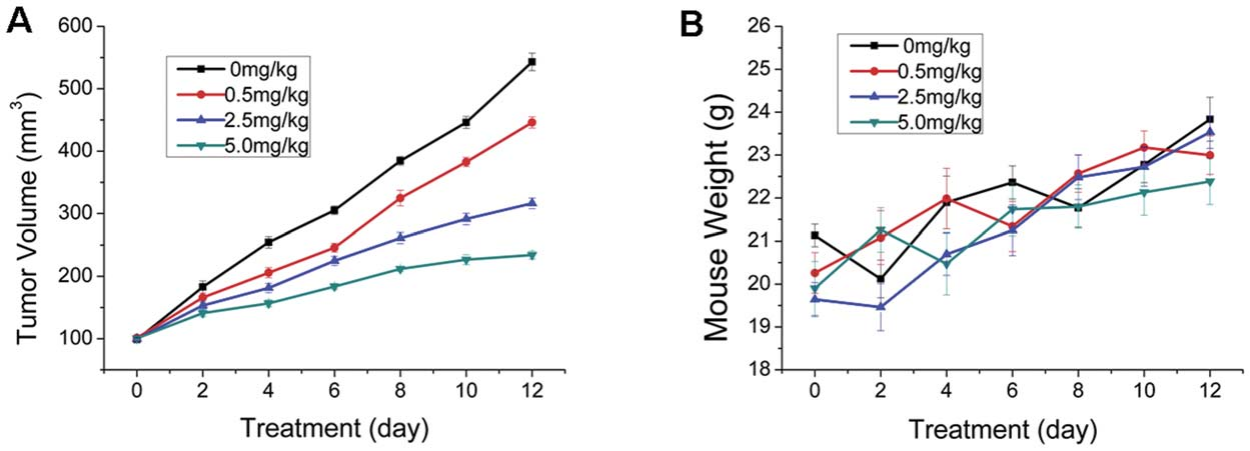
Monitoring tumor growth and body weight of tumor-bearing mice during treatment. Tumor growth (A) and body weight (B) of tumor-bearing mice treated with saline (as untreated control group), arsenic trioxide (ATO) at 0.5 mg/kg, 2.5 mg/kg or 5.0 mg/kg daily for 12 days. Six mice were used in each group. The tumor volume and body weight of all four groups were also measured every two days. Tumor volume was calculated according to the formula V = (a×b^2^)/2 where a and b represent the length and width of the tumor. Measurements were continued to 12th day. *p*<0.05 is a significant difference between control and treatment groups.

At baseline, tumor volume did not differ among the treatment groups (*p*>0.05, Figure 4A). Tumor volume growth rate on day 12, similar to tumor SI, was decreased (*p*<0.01) by 17.1%, 41.3% or 56.4% with As_2_O_3_ dosages of 0.5 mg/kg/day, 2.5 mg/kg/day or 5.0 mg/kg/day, compared with control group, respectfully. In contradistinction to the effects on tumor volume, serial As_2_O_3_ treatment did not affect body weight (*p*>0.05), suggesting no grossly apparent toxicity.

### EGFR immunohistochemical analysis

**Figure 5:**
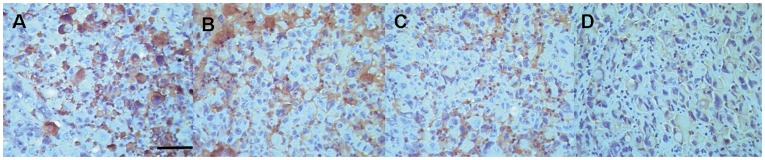
EGFR immunohistochemical assay of the tumor sections from different groups. A: 0 mg/kg As_2_O_3_ (control). The strongest red-brownish membrane-bound immunostaining on the A431 tumor tissue slice reflected the abundant over expression of EGFR (+++). B: 0.5 mg/kg As_2_O_3_ treatment group. Strong brownish membrane staining indicated plentiful of EGFR expression (++∼+++). C: 2.5 mg/kg As_2_O_3_ treatment group demonstrated moderate to low EGFR expression (+∼++). D: 5.0 mg/kg As_2_O_3_ treatment group showed weak EGFR expression (+). (Scale bar = 100 µm).

Immunohistochemical assays were carried out to correlate the magnitude of tumor uptake (signal brightness) with the tumor receptor density distribution. Tumor tissue sections from the control group exhibited the highest grade of EGFR positive staining (3+) compared with high dose treatment groups (5 mg/kg/day group, 1+; 2.5 mg/kg/day group = 1+∼2+, *p*<0.05, Figure 5), but no significant difference was noted versus the 0.5 mg/kg/day group (2+∼3+, *p*>0.05). The grade of EGFR positive stained tissue decreased as the dose of As_2_O_3_ increased, which were consistent with the *in vitro* and *in vivo* fluorescence measurements.

## Discussion

To our best knowledge, this is the first proof of concept report to demonstrate the feasibility of using near infrared optical imaging methodology to monitor the *in vivo* therapeutic effects of As_2_O_3_ on inhibiting of OSCC cells EGFR expression. The results presented here indicated that the As_2_O_3_ is active against EGFR of OSCC *in vivo* and *in vitro* and the anti-EGFR effect of As_2_O_3_ was dependent on dose and duration of exposure, which is in agreement with previous work [27].

Enthusiasm for promoting the clinical application of As_2_O_3_ (TRISENOX) has motivated the use of As_2_O_3_ in treatment of solid tumors, even though As_2_O_3_ was initially approved as an effective chemotherapeutic drug for acute promyelocytic leukemia (APL) [28]. The current widely acceptable anticancer mechanisms of As_2_O_3_ activity suggest that it induces apoptosis and influences distinct signaling pathways, including mitogen-activated protein kinases (MAPK), p53, activator protein-1 or nuclear factor kappa B [29]. Recently, other researchers have indicated that As_2_O_3_ also inhibits EGFR expression in cancer cells through p21 activation leading to cell death via the EGFR-Ras-Raf-ERK1/2 pathway based on *in vitro* or *ex vivo* methods [27], [30], however, the *in vivo* therapeutic effects of As_2_O_3_ on pharyngeal OSCC tumors and the relationship of this response to EGFR expression detected noninvasively with NIR imaging has not been explored.

As we previously demonstrated that EGF-Cy5.5 uptake into the OSCC cells was mediated by EGFR, the fluorescence signal intensity was proportional to EGFR expression of tumor cells [24]. Less intense cellular fluorescence signal was observed in treated cells compared with control cells, the fluorescent signal intensity was inversely related to As_2_O_3_ concentration (0.5 µM, 2.5 µM, or 5 µM), agreeing with IHC detection of less EGFR expression in these treated cells (Figure 1). Of note, cell numbers decreased as the arsenic trioxide concentration increased from 0 µM to 5 µM in both fluorescent and immunostained images, indicating that As_2_O_3_ induced a dose-dependent inhibition on tumor cell proliferation, consistent with previous reports [31]. In addition, EGFR expression dynamically decreased in As_2_O_3_ treatment cell groups assayed by flow cytometry, indicating the inhibiting effect of As_2_O_3_ on EGFR in dose and duration dependent manner (Figure 2). A similar phenomenon was also shown *in vivo* NIR imaging (Figure 3), which agreed with *ex vivo* EGFR expression (Figure 5). The *in vivo* imaging results were correlated with the results of *in vivo* inhibition of tumor growth by As_2_O_3_ (Figure 4A), further indicating that feasibility of using NIR optical imaging method to noninvasively monitor the therapeutic effect and the inhibiting effect of As_2_O_3_ on tumor EGFR expression *in vivo* was also dose and time dependent, consistent with previous reported research [4].

During the course of treatment, the maximum fluorescence intensity of tumors was achieved 4 h post injection of EGF-Cy5.5, which agreed with previous reports [24], [32]. Some investigators have found that fluorescence signal persisted for 4 to 5 days post injection of fluorescent agent [33], [34], but in current experiment, EGF-Cy5.5 signal was barely detectable in the tumor site 96 h post injection, with no indication of signal accumulation with serial use. This likely reflects the very low dose of contrast administered (1 nmol/kg) combined with the rapid tumor growth observed in this study.

These results have several clinical implications. Firstly, near infrared (NIR) optical molecular imaging of EGFR expression, as demonstrated in this study, can noninvasively and sensitively identify pharyngeal OSCC and can be used to longitudinally monitor and guide As_2_O_3_ treatment. While the diagnostic value of NIR fluorescent probes is frequently challenged by tissue penetration depth, despite lower background absorption, superficial neoplasms like oral squamous cell carcinoma are highly accessible to interrogation of the lesion *in situ*, providing clear advantages over biopsy and IHC for early tumor detection and for therapeutic management. Secondly, direct optical molecular imaging should provide at least semi-quantitative information regarding the spatial and temporal expression of EGFR *in vivo*. The opportunity to standardize EGFR expression level measurements would accommodate the development of improved, evidenced-based guidelines for the assessment and management of oral OSCC. In particular, noninvasive targeting imaging of early EGFR responses to medical therapy could indicate effectiveness, whereas particularly when tumor volume shrinkage is often delayed response to therapy using traditional extracellular space contrast agents [35].

Moreover, the accessibility of OSCC for direct topical or subcutaneous injection, may facilitate the use of EGF peptide–based contrast agents at lower the dosage requirements, with accelerate time to peak signal, and reduced residual contrast washout. In fact, one might envision very short interval between contrast administration and follow-up NIR examination that would readily be accommodated in the workflow patterns of dentists and oral maxillary surgeons. Finally, although no body weight loss due to As_2_O_3_ was determined in current experiment, the issue of potential toxicities with systematic administration of As_2_O_3_ remains. The oral superficial nature of OSCC suggests that direct local low dose therapy, applied topically (e.g., oral rinse) or with subcutaneous injection under image guidance would be feasible and perhaps optimal.

This study presented a proof-of-concept that noninvasive optical imaging could be used to evaluate the therapeutic effect of As_2_O_3_ by quantifying EGFR expression, likely As_2_O_3_ inhibits EGFR expression through p21 activation leading to cell death via the EGFR-Ras-Raf-ERK1/2 pathway [27], [30]. However, the linearity of the EGFR expression by tumor cells response to As_2_O_3_ therapy is not clear and will require further study to elucidate. Although useful for preliminary preclinical research, the use of the prototypical EGF-Cy5.5 agent in this study may require modification for translation. Specifically, the selection of an EGF receptor antagonist as homing ligand may be preferred to avoid activation of downstream EGFR signaling [36]. The substitution of the fluorophore Cy5.5 with a higher wavelength NIR dye would improve tissue penetration and ideally would be regulatory agency approved or approvable in order to expedite clinical experimentation [37]. Furthermore, more appropriate modeling methods accounting for variability attributable to nonspecific binding and contrast delivery efficiency (e.g., blood flow, vascular permeability, blood vessel density and hydrostatic pressure, etc) on *in vivo* receptor imaging accuracy is desirable [38].

## Conclusion

This study demonstrated that the response of EGFR expression by oral squamous carcinoma implanted within the mouse pharynx can be treated effectively with As_2_O_3_ and the response to treatment can be noninvasively assessed with EGF-Cy5.5 and NIR molecular imaging techniques. These results suggest that oral NIR molecular imaging with EGF-Cy5.5 based probes could enhance early detection as well as facilitate image based guidance for effective chemotherapeutic treatment of OSCC with As_2_O_3_.

## Materials and Methods

### Fluorochrome probe generation

The EGFR specific targeting NIR fluorescent agent, EGF-Cy5.5, was developed by coupling EGF (ImClone Systems, Branchburg, N.J., USA) to cyanine dye 5.5 (Cy5.5) molecules through a monofunctional N-hydroxysuccinimide (NHS) ester (Cy5.5-NHS, GE Healthcare, Piscataway, N.J., USA) according to our previously reported protocol [24]. Briefly, EGF (35 mg, 233.45 nmol, ImClone Systems, Branchburg, N.J., USA) was mixed with Cy5.5-NHS (4.2 mg, 1242.5 nmol, GE Healthcare, Piscataway, N.J., USA) in H_2_O (3.0 ml) in darkness at 4°C for 2 h, then the reaction was quenched by adding 3.0 ml of 5% acetic acid (HOAc). The EGF-Cy5.5 was isolated using a PD-10 disposable column (GE Healthcare, Piscataway, N.J., USA), lyophilized, and resuspended in saline at a concentration of 1 mg/ml, then stored at −20°C in darkness until use.

### *In vitro* cell studies

Human epidermoid carcinoma A431 cells (Institute of Biochemistry and Cell Biology at the Chinese Academy of Sciences, Shanghai, China) that constitutively express a high levels of EGFR [39] were obtained and maintained in Dulbecco's modified Eagle's medium (DMEM) (Invitrogen Corp., Carlsbad, CA, USA) supplemented with 10% (v/v) fetal bovine serum (FBS, Gibco BRL, Cleveland, Ohio, USA). In these series of experiments, all cells were incubated under humidified atmosphere of air/CO_2_ (19∶1) at 37°C. A431 cells were plated in flat bottomed 24-well microtiter plates on coverslips at a density of 1×10^5^ cells/well. After 24 h, cells were washed three times with phosphate buffered saline (PBS, pH 7.2), then treated with 0.0 µM, 0.5 µM, 2.5 µM, 5.0 µM As_2_O_3_ (Sigma Chemical Co., St. Louis, MO, USA) in calcium- 0.9% sodium chloride solution for either 0 h, 24 h, 48 h, or 72 h. After treatment, all cells were washed three times with PBS.

### Cell immunohistochemistry microscopy

Cellular EGFR expression was evaluated by immunohistochemistry. Briefly, cells were fixed with 4% paraformaldehyde in 4°C PBS for 25 min then washed three times with PBS. After blocking to reduce nonspecific antibody binding for 30 min, monoclonal rabbit anti-human EGFR antibody (1∶200, COOH terminus; Santa Cruz Biotechnology, Santa Cruz, Calif., USA) was incubated with the cells at 37°C for 2 h, then the unbound ligand was removed in three washes of PBS. Next, the cells were treated with a biotinylated goat anti-rabbit IgG (Southern Biotechnology Associates, Birmingham, Ala., USA), followed with a streptoavidin-biotin peroxidase reagent (Histofine kit; Nichirei Biosciences Inc., Tokyo, Japan). Finally, diaminobenzidine (DAB) and 1% hydrogen peroxidase were applied as chromogen, and the cells were counterstained with hematoxylin. Imaging was performed with a Nikon E800 microscope using a Nikon DXM 1200 digital camera (Nikon, Tokyo, Japan).

### Fluorescent microscopy assay

The treated cells were incubated with 500 µl EGF-Cy5.5 (20 nM final concentration) for 30 minutes at 37°C in darkness. After incubation, all the cells were washed three times with PBS. Fluorescence microscopy (with an Olympus microscope outfitted with NIR diode sources and filters) of tumor cells was performed for visual confirmation of EGF-Cy5.5 uptake. Diamidino-phenyl-indole (DAPI) was used to stain cell nuclei. In the microscopic images EGF-Cy5.5 was pseudo-colored red (emission at 680–710 nm), while DAPI was pseudo colored blue (emission at 461 nm).

### Flow cytometry assay

After incubation with EGF-Cy5.5, A431 cells were also suspended with trypsin solution and centrifugal elutriation twice in PBS. Quantification of fluorescent intensity of EGF-Cy5.5 binding to EGFR was assessed using flow cytometry (FACSort, Becton Dickinson, Franklin Lakes, N.J., USA). All experiments were replicated in triplicate.

### Ethics Statement

All experimental protocols were pre-approved by the Experimental Animal Ethic Committee of Harbin Medical University, China (Animal Experimental Ethical Inspection Protocol No. HAYIWEIDONGSHENZi 2010035). Use of animals was confirmed with the Guide for the Care and Use of Laboratory Animals published by the US National Institutes of Health (NIH Publication No. 85–23, revised 1996).

### *In vivo* mouse studies

Athymic nude mice (half male and half female, BALB/c-nu/nu, 4–6 weeks old, 18–22 g) from Vital River Laboratory Animal Technology Co. Ltd (National Science Institute, Beijing, China) were housed five per cage and provided with UV-sterilized pellet chow and autoclaved distilled water. Animals were maintained in a pathogen-free mouse colony at Harbin Medical University (Harbin, China). A431 cells (5×10^6^) in 200 µl were slowly injected into the floor of the mouth of anesthetized mice with isoflurane [40]. When the tumors reached 0.4 to 0.6 cm in diameter (1–2 weeks after inoculation), the tumor-bearing mice were subjected to *in vivo* NIR imaging.

### *In vivo* NIR optical imaging

To characterize EGFR expression in A431 cells *in vivo*, an eXplore Optix time-domain fluorescence imaging system (ART/GE Healthcare, Saint-Laurent, Quebec, Canada) which featured a 667-nm excitation pulse laser, with a 710-nm emission bandpass filter was used to image the tumor-bearing mice. The eXplore OPTX-optView software installed on the imaging system was used for data acquisition and processing.

The tumor-bearing mice were divided into a single control and three As_2_O_3_ treatment groups (0 mg/kg/day, 0.5 mg/kg/day, 2.5 mg/kg/day, 5.0 mg/kg/day, 6 mice/group). As_2_O_3_ was injected intraperitoneally (i.p.) on days 0, 2, 4, 6, 8, and 10. The control group received an equal volume of saline under the identical conditions. For *in vivo* characterization of EGFR, mice were sedated with isoflurane and intravenously (i.v.) injected with 1 nmol/kg EGF-Cy5.5 diluted in 0.3 ml saline via the tail vein before As_2_O_3_ treatment (on day 0) and on days 4, 8, 12 thereafter (Figure S1). Fluorescence images were acquired at 4 h post injection of EGFR targeting agent, near the peak intensity of the fluorescence signal [24], [32]. Tumor volumes and body weights of all animals were measured every 2 days, which were used as indicators of efficiency and systemic toxicity of the treatment, respectively. Tumor volume was calculated according to the formula V = (a×b^2^)/2 where a and b represent the length and width of the tumor [13]. Measurements were continued to the 12th day of study.

### Immunohistochemistry

Mice were euthanized at the termination of the study. Tumors were harvested and fixed with formalin and embedded in paraffin for immunohistochemical analysis. Tissues were sectioned at 8 µm-thickness de-paraffinized, microwave pretreated, and then incubated with 0.3% hydrogen peroxide for 30 min. EGFR labeling and analysis were accomplished by the same two-step method as described above in cell IHC methods. The immunoreactivity in tumor cells were classified and scored as follows: the intensity of staining was scored as 0, no staining (<10%); 1+, weak (10–25%); 2+, moderate (26–50%); 3+, strong (51–100%), which included at least 1000cells per sample within 5 regions of interest (ROI, 200 cells/ROI) [2], [18]. Tumor receptor density distribution analyzed by IHC was correlated the magnitude of tumor contrast uptake (i.e., signal intensity).

### Statistical analysis

Data were presented as mean ± standard deviation. The effect of time and As_2_O_3_ dose on cellular EGFR expression was statistically analyzed using a factorial design ANOVA. Fluorescence intensity was defined as total photon counts/pixel within manually inscribed region of interest (ROI) area divided by the laser pulse time (ms) and unit time [24]. The repeated-measure analysis with covariates was used to assess the effects of time and As_2_O_3_ dose on fluorescence intensity, tumor size, body weights adjusted for their baseline values, respectively. Subanalysis of specific paired comparisons utilized student's *t* test. Nonparametric tests (Mann-Whitney) were used to compare the difference of IHC results. Analyses were performed using the SPSS statistical software package (SPSS 18.0; SPSS, Inc., Chicago, Ill., USA). *P*<0.05 was considered as statistically significant.

## Supporting Information

Figure S1 **The protocol of As_2_O_3_ treatment and NIR imaging.** When the tumors reached 0.4 to 0.6 cm in diameter (1–2 weeks after inoculation), the tumor-bearing mice were divided into a single control and three As_2_O_3_ treatment groups (0 mg/kg/day, 0.5 mg/kg/day, 2.5 mg/kg/day, 5.0 mg/kg/day, 6 mice/group). As_2_O_3_ was injected intraperitoneally (i.p.) on days 0, 2, 4, 6, 8, 10 (▴). The control group was injected with an equal volume of saline under the identical conditions. For *in vivo* NIR imaging, mice were sedated with ketamine/xylazine and intravenously (i.v.) injected with 1 nmol/kg EGF-Cy5.5 diluted in 0.3 ml saline via the tail vein before As_2_O_3_ treatment (on day 0) and on days 4, 8, 12 (▪) after As_2_O_3_ treatment. (The arrow indicated the tumor area).
(TIF)
