# Supplementary figures and images for: Near Infrared Imaging of EGFR of Oral Squamous Cell Carcinoma in Mice Administered Arsenic Trioxide
Source: PLoS One. 2012 Sep 28;7(9):e46255. doi: 10.1371/journal.pone.0046255 (PMC3460885; doi:10.1371/journal.pone.0046255)

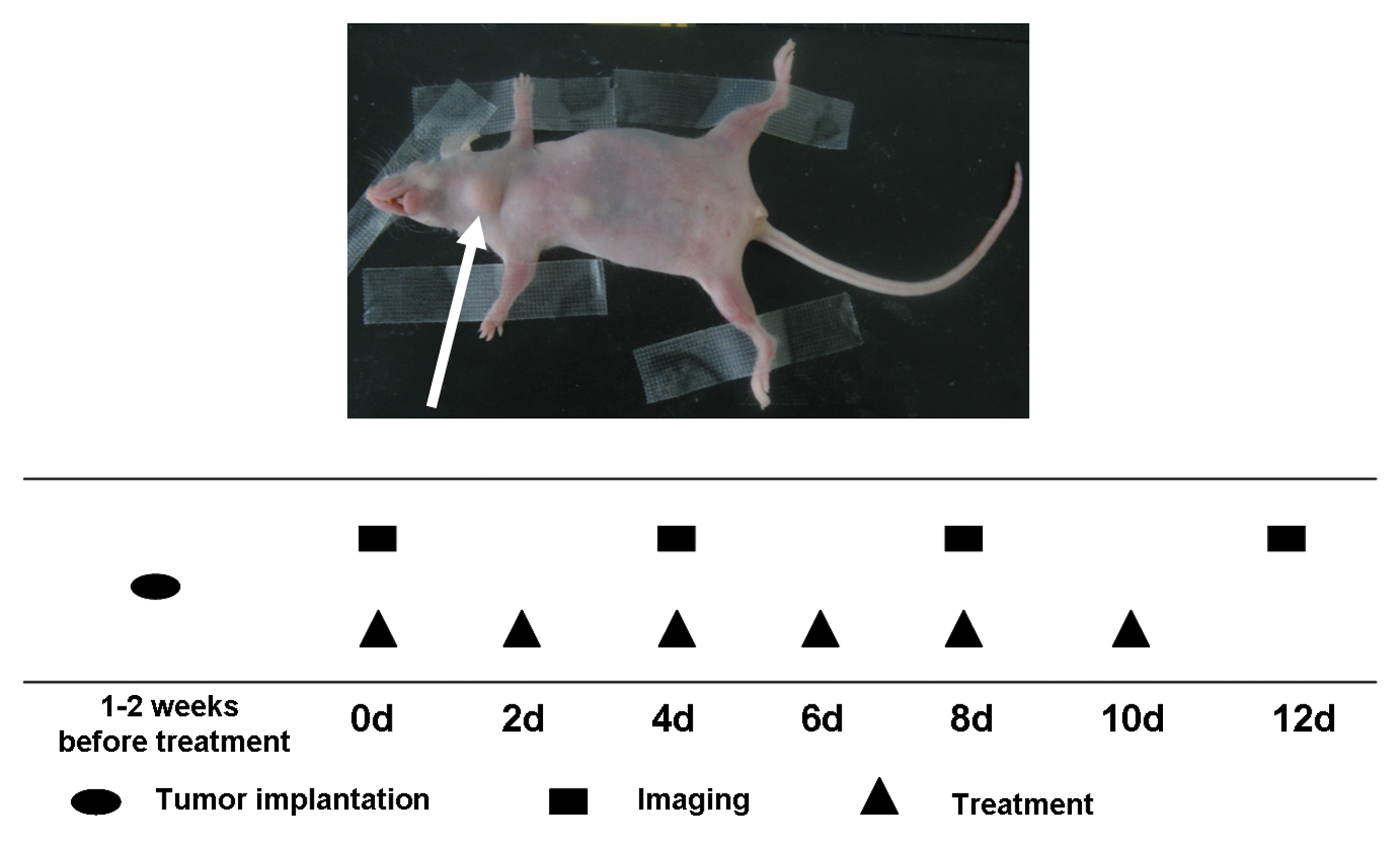

Supplement: Figure S1 — The protocol of As2O3 treatment and NIR imaging. When the tumors reached 0.4 to 0.6 cm in diameter (1–2 weeks after inoculation), the tumor-bearing mice were divided into a single control and three As2O3 treatment groups (0 mg/kg/day, 0.5 mg/kg/day, 2.5 mg/kg/day, 5.0 mg/kg/day, 6 mice/group). As2O3 was injected intraperitoneally (i.p.) on days 0, 2, 4, 6, 8, 10 (▴). The control group was injected with an equal volume of saline under the identical conditions. For in vivo NIR imaging, mice were sedated with ketamine/xylazine and intravenously (i.v.) injected with 1 nmol/kg EGF-Cy5.5 diluted in 0.3 ml saline via the tail vein before As2O3 treatment (on day 0) and on days 4, 8, 12 (▪) after As2O3 treatment. (The arrow indicated the tumor area). (TIF) [file pone.0046255.s001.tif]
